# Genotyping Cancer-Associated Genes in Chordoma Identifies Mutations in Oncogenes and Areas of Chromosomal Loss Involving CDKN2A, PTEN, and SMARCB1

**DOI:** 10.1371/journal.pone.0101283

**Published:** 2014-07-01

**Authors:** Edwin Choy, Laura E. MacConaill, Gregory M. Cote, Long P. Le, Jacson K. Shen, Gunnlaugur P. Nielsen, Anthony J. Iafrate, Levi A. Garraway, Francis J. Hornicek, Zhenfeng Duan

**Affiliations:** 1 Division of Hematology Oncology, Massachusetts General Hospital, Boston, Massachusetts, United States of America; 2 Sarcoma Biology Laboratory, Center for Sarcoma and Connective Tissue Oncology, Massachusetts General Hospital, Boston, Massachusetts, United States of America; 3 Center for Cancer Genome Discovery and Department of Medical Oncology, Dana Farber Cancer Institute, Harvard Medical School, Boston, Massachusetts, United States of America; 4 Department of Pathology, Massachusetts General Hospital, Boston, Massachusetts, United States of America; University of Pittsburgh Cancer Institute, United States of America

## Abstract

The molecular mechanisms underlying chordoma pathogenesis are unknown. We therefore sought to identify novel mutations to better understand chordoma biology and to potentially identify therapeutic targets. Given the relatively high costs of whole genome sequencing, we performed a focused genetic analysis using matrix-assisted laser desorption/ionization-time of flight mass spectrometer (Sequenom iPLEX genotyping). We tested 865 hotspot mutations in 111 oncogenes and selected tumor suppressor genes (OncoMap v. 3.0) of 45 human chordoma tumor samples. Of the analyzed samples, seven were identified with at least one mutation. Six of these were from fresh frozen samples, and one was from a paraffin embedded sample. These observations were validated using an independent platform using homogeneous mass extend MALDI-TOF (Sequenom hME Genotyping). These genetic alterations include: ALK (A877S), CTNNB1 (T41A), NRAS (Q61R), PIK3CA (E545K), PTEN (R130), CDKN2A (R58*), and SMARCB1 (R40*). This study reports on the largest comprehensive mutational analysis of chordomas performed to date. To focus on mutations that have the greatest chance of clinical relevance, we tested only oncogenes and tumor suppressor genes that have been previously implicated in the tumorigenesis of more common malignancies. We identified rare genetic changes that may have functional significance to the underlying biology and potential therapeutics for chordomas. Mutations in CDKN2A and PTEN occurred in areas of chromosomal copy loss. When this data is paired with the studies showing 18 of 21 chordoma samples displaying copy loss at the locus for CDKN2A, 17 of 21 chordoma samples displaying copy loss at PTEN, and 3 of 4 chordoma samples displaying deletion at the SMARCB1 locus, we can infer that a loss of heterozygosity at these three loci may play a significant role in chordoma pathogenesis.

## Introduction

Chordoma is an aggressive primary malignancy of the axial skeleton that is thought to originate from notochordal tissue[1]. The majority of these tumors occur in either the base of the skull (35%) or in the sacrum (50%), and these are typically managed with surgery and/or radiation[2]–[10]. Their clinical behavior is marked by slow growth and a predilection to recur despite surgical resection. Most patients with recurrent chordoma and almost all patients with metastasis will eventually die from this disease[11], [12]. Unfortunately, there is no effective systemic agent to control unresectable or metastatic chordoma and the development of new treatments is limited by our poor understanding of its biology[12]–[15].

The identification of driver mutations in malignancy, such as EGFR-mutated lung cancers, c-Kit mutated gastrointestinal stromal tumors, and ALK-translocated tumors, among others, has dramatically changed the treatment landscape for these diseases [15]–[20]. We hypothesize, therefore, that for a typically chemoresistant tumor such as chordoma where there is no effective systemic therapy, the identification of mutations in genes for which there exist a therapeutic strategy can inform the development of novel drugs. However, because of the rarity of patients with these tumors, comprehensive molecular understanding of chordoma pathogenesis is currently incomplete.

Karyotype studies observed chordomas with loss at chromosome 1p and 3p and gains involving chromosomes 7q, 20, 5q, and 12q [21], [22]. Mobley et al. identified a cohort of poorly differentiated chordomas that lack SMARCB1/INI1 expression [23]. Cytogenetic evaluation using FISH showed that 3 of the 4 samples had deletion at this locus. Gene sequencing did not reveal any point mutations. Recently, Le et al. performed comparative genomic hybridization on 21 chordoma tumor samples to show frequent loss of chromosomal regions 9p21 and 10q23.3 [24]. They also genotyped for 56 point mutations occurring in 13 genes commonly associated with cancer, including CDKN2A (located in 9p21) and PTEN (located in 10q23.3) but did not identify any alterations in their cohort. We sought to expand on these observations by analyzing 45 chordoma samples via high throughput genotyping of cancer-associated genes known to play an important role in cancer.

## Methods

### Ethics Statement

Institutional review board approval was obtained to retrospectively study these tumor samples from the Partners Human Research Committee, 116 Huntington Avenue, Suite 1002, Boston, MA 02116. The protocol number is 2007P-002464. The institutional review board waived the need to consent. Tumor specimens were obtained from the clinical archives of Dr. Francis Hornicek (Department of Orthopaedic Surgery, Massachusetts General Hospital) and the Massachusetts General Hospital Tissue Repository (http://www.massgeneral.org/cancer/research/resourcelab.aspx?id=31).

### Chordoma tumor samples

Tumor specimens were obtained from the clinical archives of Dr. Francis Hornicek (Department of Orthopaedic Surgery, Massachusetts General Hospital) and the Massachusetts General Hospital Tissue Repository (http://www.massgeneral.org/cancer/research/resourcelab.aspx?id=31).

### Extraction of Genomic DNA

Extraction of DNA from chordoma tumor tissues and cell lines were performed using QIAamp DNA Micro kit (Qiagen) as previously described in prior publications [25]. The extraction was carried out according to the manufacturer's instructions. DNA was preserved at −20°C until use.

### Selection of Cancer Gene Mutations and OncoMap v3 Assay Design

Selection of cancer gene mutations for assay design and mass spectrometric genotyping were performed as previously described [25]–[27]. Multiple databases, including the Sanger Institute COSMIC database, PubMed, and The Cancer Genome Atlas (TCGA), were queried for known somatic oncogene and tumor suppressor gene mutations. Mutations were ranked in importance by frequency in cancers and across cancer subtypes, and genes with existing therapeutic modifiers were preferentially selected.

Primers for PCR amplification and the extension probe were designed using Sequenom MassARRAY Assay Design 3.0 and the resulting DNA sequences were (1) queried in the dbSNP database to avoid incorporation of SNPs during assay design, (2) confirmed through a BLAST-like alignment tool (BLAT) and modified where necessary to avoid pseudogene amplification[28], and (3) synthesized unmodified using standard purification (Integrated DNA Technologies, Coralville, IA).

### Mass Spectrometric Genotyping

Primers and probes were pooled and then validated on control DNA derived from the CEPH panel of human HapMap DNAs (Coriell Institute) as well as a panel of human cell lines with known mutational status, as described previously[26]. Genomic DNA was quantified using Quant-iT PicoGreen dsDNA Assay Kit (Invitrogen, Carlsbad, California), subjected to whole-genome amplification (WGA) using Qiagen Repli-g kit, and a post-WGA cleanup step was implemented using a Nucleofast Purification Kit (Macherey-Nagel). Mass spectrometric genotyping using iPLEX chemistries was performed as previously published [27].

Candidate mutations were further filtered by manual review and selected for confirmation using multi-base extension homogenous Mass-Extend (hME) chemistry with plexing of ≤6 assays per pool. Conditions for hME validation were consistent with the methods described by MacConaill et al. 2009 [27].

### Array Comparative Genomic Hybridization (CGH) Analysis

Array CGH using the Agilent 4x180k CGH + SNP microarray (Santa Clara, CA) was previously described [24]. Copy number aberration calls were made with a minimum regional absolute average log base 2 ratio of 0.25 and minimum contiguous probe count of 5. All array data were manually reviewed for subtle changes.

## Results

### Characteristics of Clinical Tumor Samples

A total of 45 DNA samples were derived from 40 patients who had undergone operative resections of their chordoma. Patient date of surgery (DOS), age, sex, tumor location, recurrence, and metastasis are catalogued in Table 1. 28 specimens were obtained from fresh frozen tissue, and 17 were derived from FFPE blocks.

**Table 1:** Characteristics of Tumor Samples.

Clinical data of DNA samples from chordoma tissues							
Patient	Date of Surgery	Age	Sex	Metastatic	Recurrent	Location	Mutation
1	12/23/1994	60	M	No	Yes	Sacrum	
2	9/22/1995	35	M	Yes	No	L1–L2	
3	10/2/1996	77	M	No	No	Sacrum	
4	10/30/1996	74	M	No	Yes	Sacrum	*PIK3CA*E545K
5	12/20/1996	55	M	No	No	Sacrum	
6	1/3/1997	73	M	No	Yes	Sacrum	
7	1/7/1997	58	M	Yes	No	Left scapula	*CDKN2A* R58* *SMARCB1* R40*
8	3/27/1998	60	M	Yes	No	Chest wall	*CDKN2A* R58* *SMARCB1* R40*
9	2/3/1999	48	M	No	No	Sacrum	*CTNNB1* T41A
10	5/12/1999	62	M	No	No	Sacrum	
11	2/11/2000	52	F	No	Yes	L3	
12	6/26/2000	63	M	No	No	Sacrum	*PTEN* R130*
13	10/18/2000	51	M	Yes	No	Sacrum	
14	11/22/2000	71	F	No	No	L4	
15	4/25/2001	50	F	No	No	Sacrum	
16	5/16/2001	46	M	No	No	Sacrum	
17	7/25/2001	74	M	No	Yes	Sacrum	
18	9/19/2001	70	M	No	Yes	Sacrum	*NRAS* Q61R
19	4/12/2002	73	M	No	Yes	Sacrum	
20	10/30/2002	83	M	No	Yes	Sacrum	
21	12/6/2002	37	M	No	Yes	Sacrum	
22	1/15/2003	75	M	No	Yes	Sacrum	
23	5/20/2003	74	M	No	Yes	Sacrum	
24	1/30/2004	64	M	No	No	Sacrum	
25	12/14/2004	60	F	No	No	Sacrum	
26	5/3/2006	50	M	No	Yes	T12–L1	
27	7/5/2006	52	F	No	Yes	Sacrum	
28	8/3/2007	71	M	No	No	Sacrum	
29	5/16/2001	46	M	No	No	Sacrum	
30	8/16/2002	80	M	No	No	Sacrum	
31	N/A	58	F	No		Clivus	
32	2/14/2007	53	M	No	No	Skull base	
33	12/14/2004	60	F	No	No	Sacrum	
34	7/29/1993	41	M	No	Yes	Lumbar	
35	4/12/2002	73	M	No	Yes	Sacrum	
36	5/5/2006	70	M	No	Yes	Sacrum	
37	9/6/2006	73	M	No	No	T11	
38	6/26/2000	63	M	No	No	Sacrum	
39	7/30/2003	52	F	No	No	Sacrum	*ALK* A877S
40	10/2/1996	77	M	No	No	Sacrum	
41	8/3/2007	71	M	No	No	Sacrum	
42	8/4/1999	52	M	No	Yes	Lumbar	
43	5/21/2003	74	M	No	Yes	Sacrum	
44	1/6/2004	71	F	No	No	Cervical	
45	12/20/1996	55	M	No	No	Sacrum	

Samples 1–28 derived from fresh frozen tissue; Samples 29–45 derived from formalin fixed paraffin embedded tissue.

### Genotype and CGH Analysis

**Figure 1:**
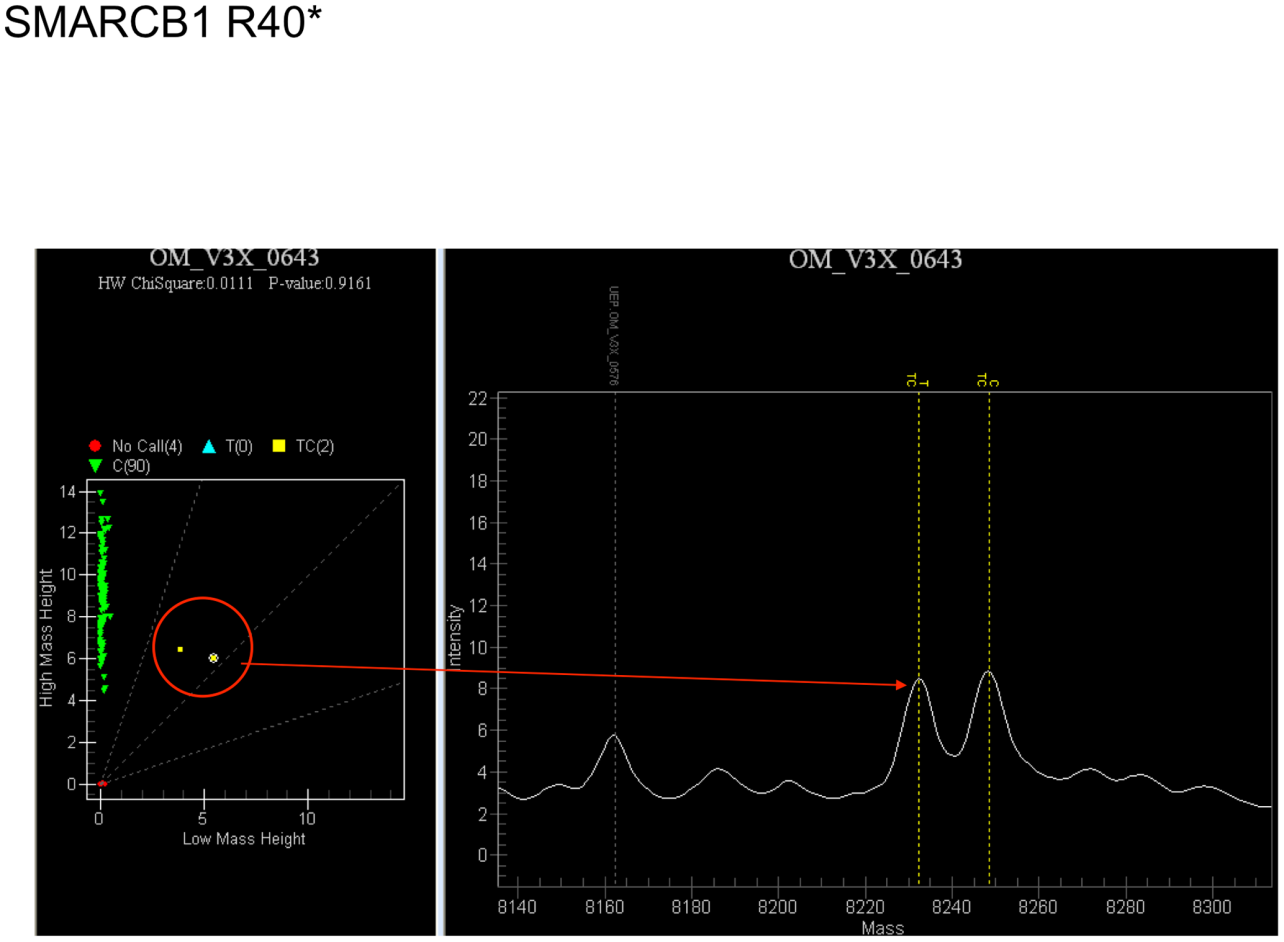
Mass Spectrophotometry Plot: Observing a Mutation in SMARCB1. PCR products differ in mass depending on whether the probe extension detects a cytosine (C) or thymine (T). The figure in the right shows mass spec plot of an allele in SMARCB1 displaying a peak at both C and T. The left panel shows that most samples tested were homozygous with C, and 2 samples were heterozygous.

Examples of the mass spectrometric readouts are shown in Figure 1. Mutation results are catalogued alongside patient characteristics in Table 1. The identified mutations include: ALK (A877S), CTNNB1 (T41A) also known as β-Catenin, NRAS (Q61R), PIK3CA (E545K), PTEN (R130), CDKN2A (R58*), and SMARCB1 (R40*).

**Figure 2:**
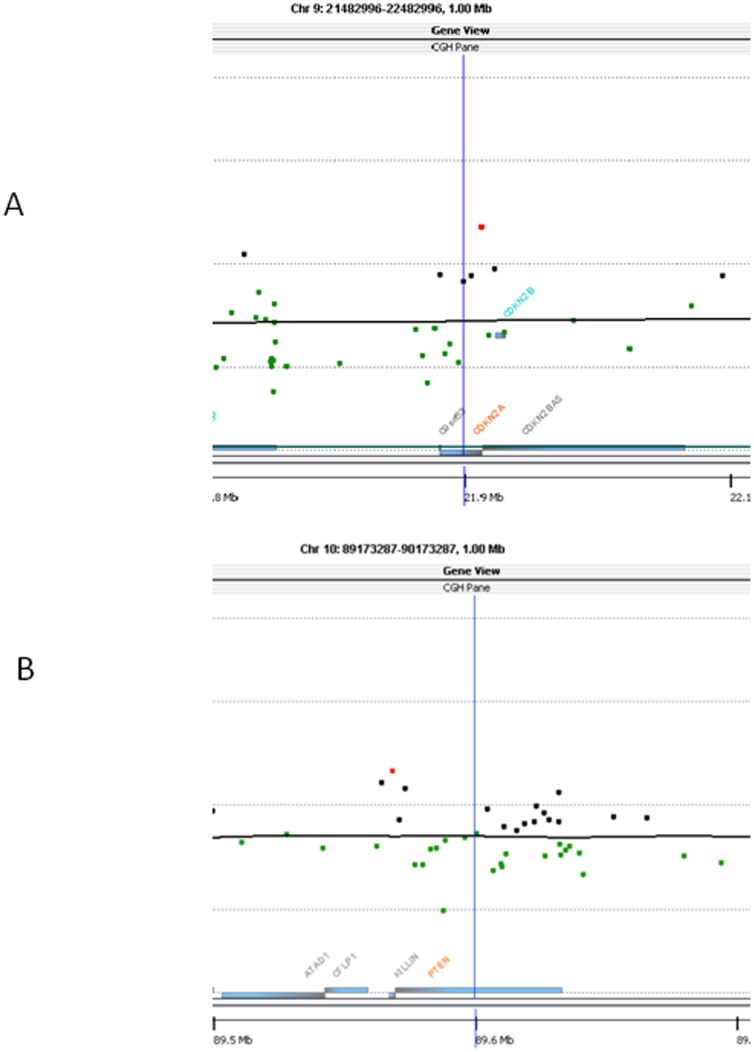
Selected View of aCGH Analyses (Chromosomes 9 and 10) from Two Cases. X-axis measures location along chromosome 9 (Panel A) or 10 (Panel B). Y-axis measures relative probe count. **A: aCGH showing heterozyogous copy loss at CDKN2A; B: aCGH showing heterozyogous copy loss at PTEN.**

Among the 28 freshly frozen samples tested, 6 mutations were identified. Of the 17 FFPE samples tested, only 1 mutation (in ALK) was identified. Similar to our previously reported rates of detecting similar frequency of mutations between freshly frozen and paraffin embedded osteosarcoma tumor samples [25], there appeared to be no difference in the rate of mutations in samples that were freshly frozen when compared to the samples of FFPE prepared (Fisher's exact test, p = 0.22 two tails). Two samples (samples #7 and 8 in Table 1 ), taken from the same patient at different anatomic locations and surgical dates, each had the same mutations in both SMARCB1 and CDKN2A. When we performed CGH analysis on the samples with CDKN2A and PTEN mutations, we observed copy number losses at respective loci (Figure 2), demonstrating that these mutations occurred in regions of chromosomal loss.

## Discussion

Chordomas have been previously described to have chromosomal aberrations and are characterized by chromosomal gains and losses at various regions throughout the genome [21], [22], [24], [29]. A comprehensive genome-wide survey for high-yield mutations have not yet been performed across a large collection of chordomas. Array comparative genomic hybridization (aCGH) was used to analyze 21 chordoma tumor samples[14] and observed large copy number losses involving chromosomes 1p, 3, 4, 9, 10, 13, 14, and 18 [24]. In that study, a focused analysis of 11 cancer-related genes did not reveal any new mutations in DNA sequence. Diaz et al. performed a whole-genome single-nucleotide polymorphism microarray analysis of 21 clival chordoma samples to confirm CGH findings by others that chromosome 3 aneuploidy and chromosome 9p deletion occurs (albeit less frequently than in sacral chordomas)[29].

With current technology, whole-genome sequencing of large collections of chordomas is costly and laborious. Thus, we aimed to broaden our mutational screen while focusing only on those genetic alterations that have been previously implicated as tumorigenisis drivers in other tumor types with the overall goal of identifying new mutations that could direct further research into chordoma therapeutic discovery. Some of the mutations identified in this study are in genes well-known for their role as tumor suppressors, such as PTEN and CDKN2A. Interestingly, both of these genes lie in chromosomal regions that were frequently found to have copy number losses in the recent CGH array analysis (9p21 for CDKN2A and 10q23.3 for PTEN) at a rate of >80% for each. Therefore, we performed CGH in the samples containing mutations in PTEN and CDKN2A and found chromosomal loss at each loci in the respective samples. Interestingly, 33% of samples tested in Le et al. collection found complete loss of both copies of CDKN2A – arguing that either a point mutation at CDKN2A or complete copy loss of CDKN2A can lead to LOH at this locus [24]. We therefore infer that loss of heterozygosity (LOH) at these loci can potentially be a tumorigenic event for the development of chordoma.

SMARCB1 is a subunit of the ATP-dependent chromatin remodeling complex SWI/SNF, a powerful epigenetic tumor suppressor, which directly antagonizes the histone methyltransferase EZH2. Mutations in SWI/SNF members are increasingly being recognized in malignancies where they are now thought by some groups to be more common than inactivating mutations in p53 [30], [31]. SMARCB1 also regulates the cell-cycle by activating CDKN2A, and its loss leads to upregulation of EZH2 a molecule that is being targeted by several inhibitors that are currently undergoing clinical testing [32]–[34]. SMARCB1 is known to be disrupted in malignant rhabdoid tumors, round cell soft-tissue sarcomas (most were a subset of tumors resembling extraskeletal myxoid chondrosarcoma with rhabdoid features, epithelioid sarcomas, and schwannomatosis [34]–[38].

These tumors, like chordomas, are groups of rare neoplasms for which no effective therapy is known. Mobley et al. observed that in poorly differentiated chordomas, expression of SMARCB1 is also lacking. They used FISH to evaluate the SMARCB1 locus at chromosome 22q and found deletion in 3 of 4 samples[38]. We therefore also infer that when SMARCB1 mutations coincide in areas of chromosomal loss, loss of heterozygosity can also be a tumorigenic event.

Several of the mutations found in our study, such as ALK, PIK3CA, and CTNNB1, each have inhibitors that are either commercially available, such as the ALK inhibitor XALKORI (crizotinib), or have multiple inhibitors currently under active clinical development[39]–[44]. Observations such as ours suggest the clinical utility of performing a genotype directed clinical trial to test new kinase inhibitors in this disease population.

In conclusion, we observed chordomas with point mutations in tumor suppressor genes in areas of frequent chromosomal loss and in oncogenes with commercially available inhibitors. The former suggests possible mechanisms for chordoma tumorigenesis, the latter suggests possibilities for novel therapies. Further studies need to be done in order to establish these genes as important biomarkers or targets in chordoma. The ideal study would be a complete genome sequencing effort involving many more chordoma samples. We hope that this initial sign of successful genotyping in the largest collection of human chordoma tumor samples to date may ignite enthusiasm to further investigations into the molecular pathology of this disease that currently has no effective medical therapy.
